# Impact of Doxorubicin on Cell-Substrate Topology

**DOI:** 10.3390/ijms23116277

**Published:** 2022-06-03

**Authors:** Andreas Krecsir, Verena Richter, Michael Wagner, Herbert Schneckenburger

**Affiliations:** Institute of Applied Research, Aalen University, 73430 Aalen, Germany; andreas.krecsir@studmail.htw-aalen.de (A.K.); richter_v@web.de (V.R.); michael.wagner@hs-aalen.de (M.W.)

**Keywords:** fluorescence imaging, doxorubicin, TIRFM, cell-substrate distances, apoptosis

## Abstract

Variable-Angle Total Internal Reflection Fluorescence Microscopy (VA-TIRFM) is applied in view of early detection of cellular responses to the cytostatic drug doxorubicin. Therefore, we determined cell-substrate topology of cultivated CHO cells transfected with a membrane-associated Green Fluorescent Protein (GFP) in the nanometer range prior to and subsequent to the application of doxorubicin. Cell-substrate distances increased up to a factor of 2 after 24 h of application. A reduction of these distances by again a factor 2 was observed upon cell aging, and an influence of the cultivation time is presently discussed. Applicability of VA-TIRFM was supported by measurements of MCF-7 breast cancer cells after membrane staining and incubation with doxorubicin, when cell-substrate distances increased again by a factor ≥ 2. So far, our method needs well-defined cell ages and staining of cell membranes or transfection with GFP or related molecules. Use of intrinsic fluorescence or even light-scattering methods to various cancer cell lines could make this method more universal in the future, e.g., in the context of early detection of apoptosis.

## 1. Introduction

Doxorubicin, an anthracycline antibiotic, is used as a cytostatic drug in cancer chemotherapy, such as breast cancer, bronchial carcinoma, and lymphoma, and has been studied and applied for several decades [1,2]. The drug is taken up by cells either by passive diffusion through their membrane [3] or by endocytosis after encapsulation [4,5] and finally intercalates in DNA strands, where it causes chromatin condensation and initiates apoptosis [6,7]. In cardiomyocytes, densely packed with mitochondria, oxidative stress is a key factor during treatment with anthracycline dyes [8], which are used in chemotherapy or heart failure [9]. Due to its fluorescence properties [10], doxorubicin can be localized within the cells, e.g., by wide-field microscopy, fluorescence lifetime measurements [11,12,13], or hyperspectral imaging [14].

While in previous papers we focused on the uptake and intracellular distribution of doxorubicin in 2-dimensional [15] and 3-dimensional [16] cell cultures, we now draw our attention to the plasma membrane, in particular to cell-substrate topology, in order to obtain new information on cell morphology upon application of doxorubicin. Chinese Hamster Ovary (CHO) cells were transfected with a membrane-associated Green Fluorescent Protein (GFP), and the distance between the fluorescent cell membrane and a glass slide, upon which cells were growing as monolayers, was calculated for all pixels from individual images acquired by Variable-Angle Total Internal Reflection Fluorescence Microscopy (VA-TIRFM) [17]. This technique was used in order to measure early cellular responses caused by doxorubicin within 2–24 h, since conventional tests proved cytotoxicity only after an incubation time t ≥ 24 h for 2D cell cultures [18,19,20] and after t = 48–96 h for 3D cultures [16]. In addition, established cytotoxicity tests require considerably more time for evaluation (up to about 7 days for a colony formation assay [21]).

## 2. Theory

Our experiments are based on Variable-Angle Total Internal Reflection Microscopy (VA-TIRFM) with light incidence on a cell monolayer at an angle Θ, which is larger than the critical angle Θc = arcsin (n_2_/n_1_) for total internal reflection (TIR) with n_1_ corresponding to the refractive index of the medium of light incidence and n_2_ (≤n_1_) to that of the cells. Using a two-layer model with refractive indices of a glass prism (n_1_) and the cytoplasm (n_2_), an evanescent electromagnetic field arises on the cell-substrate interface with an exponential decrease and a penetration depth [22]

d(Θ) = (λ/4π) (n_1_^2^ sin^2^Θ − n_2_^2^)^−1/2^ (1)

The refractive index of the plasma membrane is negligible in this equation due to its thickness of around 5 nm. A previous calculation [17] showed that the fluorescence intensity of a fluorophore located in a thin layer of thickness t (e.g., cell membrane) and excited by the evanescent field can be approximated as

I_F_ = A c T(Θ) t e^−^^Δ^/d(Θ) (2)

with an experimental constant A, the concentration c of the fluorophore, the transmission factor T between media of different refractive indices, and the distance Δ between the cell membrane and the substrate (see also Supplementary Information, Section S2). With

T(Θ) = 4cos^2^ Θ/[1 − n_2_/n_1_)^2^] (3)

for polarized light perpendicular to the plane of incidence [23], a plot of ln[I_F_/T(Θ)] over 1/d(Θ) results in a linear function with the slope −Δ according to Equation (2). Δ corresponds to the cell-substrate distance of each pixel, as further visualized in [24]. In the present paper, Δ was evaluated from fluorescence images recorded under VA-TIRFM.

## 3. Results

**Figure 1:**
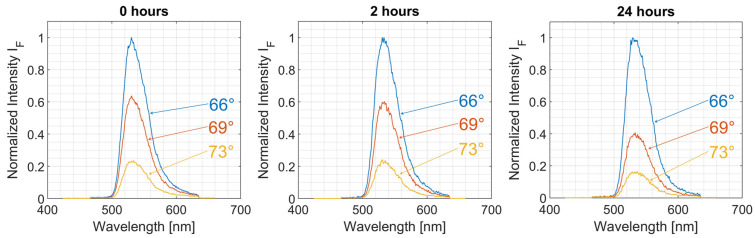
Fluorescence spectra of CHO-pAcGFP1-Mem cells at varying TIR angles after 0 h, 2 h, and 24 h incubation with doxorubicin (2 µM) in culture medium. Subcultures with 56–64 cell splittings.

Representative fluorescence spectra from subcultures with 56–64 cell splittings are depicted in Figure 1 for Total Internal Reflection (TIR) angles of 66°, 69°, and 73° after 72 h cell growth and (subsequently) 0 h (control), 2 h, or 24 h incubation with doxorubicin in cultivation medium. The spectra are characteristic for Green Fluorescent Protein (GFP) with emission maxima around 530 nm, although some overlap by the fluorescence of intracellular doxorubicin or its degradation products, e.g., 7,8-dehydro-9,10-desacetyldoxorubicinone [25], cannot be excluded. At increased incubation times, the fluorescence signal shows a stronger decrease with an increasing angle of incidence, i.e., with a decreasing penetration depth of the evanescent field (see also Supplementary Material, Figure S1).

**Figure 2:**
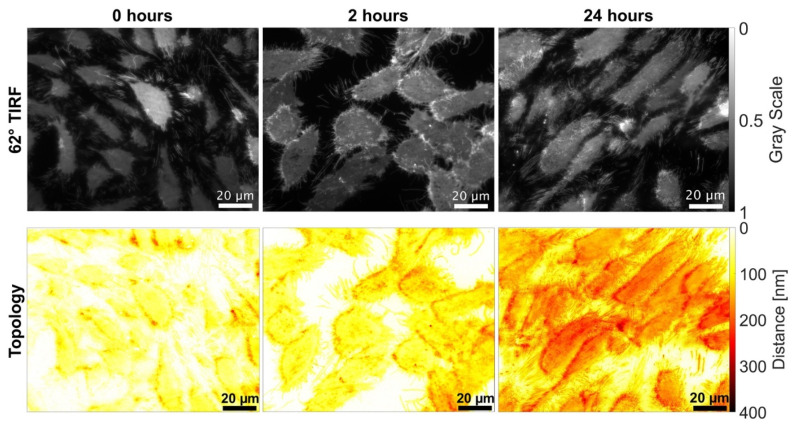
Fluorescence images at Θ = 66° excitation (upper part) and cell-substrate topology (lower part) for 0 h, 2 h, and 24 h incubation with doxorubicin (2 µM) displayed in a color code. CHO-pAcGFP1-Mem cells of subcultures 56–64 after 72 h (left, middle) or 96 h (right) growth in culture medium.

This result is confirmed by Figure 2, which shows representative fluorescence images at 66° excitation as well as the cell-substrate topology calculated for 0 h, 2 h, and 24 h incubation with doxorubicin according to Equation (2). In addition to the increasing density of cells at 24 h (due to the longer growth time), the images of cell topology show larger cell-substrate distances with increasing incubation time.

**Figure 3:**
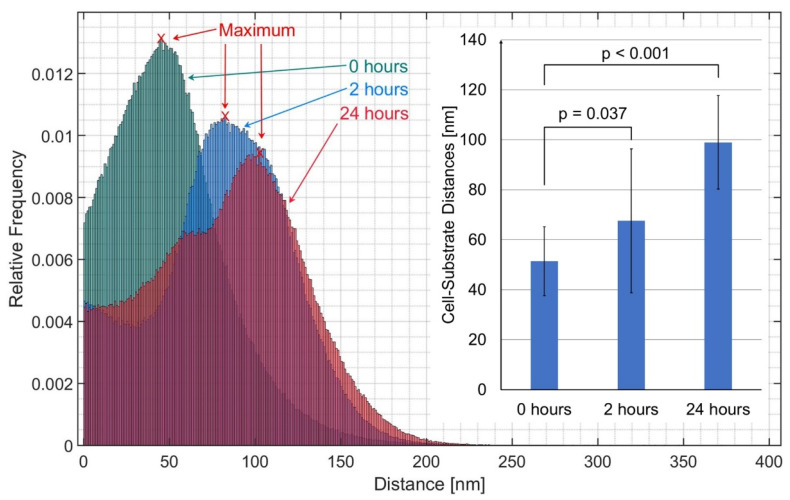
Histograms of cell-substrate distances for representative TIRFM images of CHO-pAcGFP1-Mem cells after 0 h, 2 h, and 24 h incubation with doxorubicin (2 µM) in cultivation medium. The value at 0 nm (resulting from outside the cells) has been omitted. Inset: most frequent distances evaluated from all histograms as mean value ± standard deviation and *p*-values for statistical significance obtained from a *t*-test for 2 samples assuming unequal variances (*p* ≤ 0.05: statistically significant). Subcultures with 56–64 cell splittings.

In Figure 3, the result is quantified in the histograms showing the frequencies of cell-substrate distances of representative images calculated after 0 h, 2 h, and 24 h incubation (zero distances calculated for regions outside the cells are omitted). Furthermore, the position of the maximum, i.e., the most frequent distance, is indicated. This distance—evaluated for all the histograms—was (50 ± 14) nm for 0 h, (67 ± 28) nm for 2 h, and (99 ± 20) nm for 24 h incubation, as depicted in the inset in Figure 3. According to a *t*-test for unequal variances, differences between 0 h and 2 h as well as between 0 h and 24 h were statistically significant. In an additional experiment, cells were grown for 96 h in culture medium, and most frequent cell-substrate distances were evaluated in a similar way, resulting in (50 ± 10) nm without and (82 ± 5) nm after 2 h incubation with doxorubicin. Values without doxorubicin were thus shown to be independent from growth time in culture, and values after 2 h incubation with doxorubicin differed only slightly in a statistically nonsignificant way.

**Figure 4:**
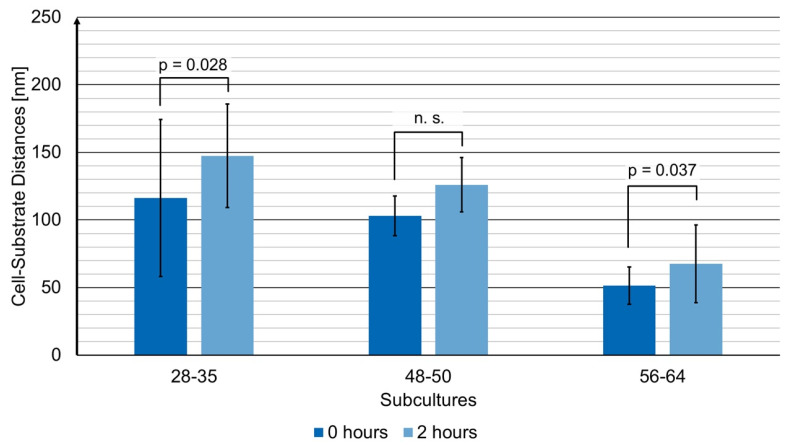
Most frequent cell-substrate distances evaluated from all histograms of the subcultures with 28–35, 48–50, and 56–64 cell splittings as mean value ± standard deviation including the *p*-values for statistical significance (*p* ≤ 0.05: statistically significant; n.s.: nonsignificant). Results are shown for 0 h and 2 h incubation with doxorubicin (2 µM).

Figure 4 shows a comparison of cell-substrate distances (most frequent distances determined from the histograms as mean value ± standard deviation) for the subcultures with 28–35, 48–50, and 56–64 cell splittings for 0 h and 2 h incubation with doxorubicin. In comparison with subcultures 56–64 (previously presented in detail), the younger cell cultures generally showed higher cell-substrate distances, which increased after incubation with doxorubicin (subcultures 28–35 from 116 ± 58 nm to 147 ± 38 nm and subcultures 48–50 from 103 ± 14 nm to 126 ± 20 nm). The relative increase was similar to that of subcultures 56–64, but only the increase for subcultures with 28–35 and 56–64 cell splittings was statistically significant (levels of significance: *p* ≤ 0.05 between 0 h and 2 h in both cases; *p* ≤ 0.001 between 0 h and 24 h for subcultures 56–64). The increase for subcultures 48–50 may be regarded as a relevant trend, which could not be tested for statistical significance due to the low number of individual measurements (*n* = 6 each).

## 4. Discussion

The measurements described above show increasing cell-substrate distances upon incubation with doxorubicin for *t* ≥ 2 h. Since this time is shorter than incubation times generally used in cytotoxicity tests, our method appears applicable for early detection of cellular responses to doxorubicin. However, with a view to validate our method, some open questions remain to be resolved: are there any additional effects due to the retention time of the cells in culture medium (without any drug), e.g., due to stiffening of microtubules [26] or cellular traction forces [27]? Previous findings of CHO-K1 cells expressing a human insulin receptor (hIR) and glucose transporter 4-myc-GFP [28] proved that growth time of the cells in a culture medium may have an impact on cell-substrate topology. In the present case, prolongation of the cultivation time from 72 h to 96 h did not change the cell-substrate distances, and after incubation of the cells for 2 h with doxorubicin, these distances were only slightly, but not significantly, larger if a growth time of 96 h instead of 72 h was chosen. Therefore, only further experiments with a larger amount of data can finally settle this question. Furthermore, the age of the cell cultures seems to play a major role. In the present case, we primarily evaluated subcultures with 56–64 splittings, i.e., rather aging cells. When, in additional experiments, we evaluated subcultures with 28–35 or 48–50 cell splittings, the cell-substrate distances were higher by about a factor of 2, while the relative increase after 2 h incubation with doxorubicin was similar to that of the subculture with 56–64 splittings, as shown in Figure 4. This implies that changes in cell-substrate distances may still be regarded as an early response to doxorubicin, but for further evaluation, cell ages should be well-defined. Cell aging may have an impact on the mechanical properties of the cell, e.g., due to mechano-transduction and modified tension of the cytoskeleton (for a review, see [29]) or due to an altered stiffness of cell membranes in connection with a changing cholesterol level [30].

**Figure 5:**
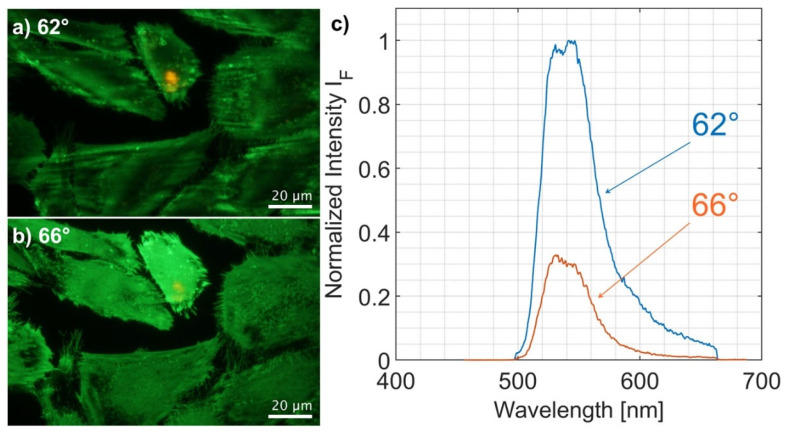
Fluorescence images (**a**,**b**) and fluorescence spectra (**c**) of CHO-pAcGFP1-Mem cells 24 h after incubation with doxorubicin for Θ = 62° (whole cell excitation) and Θ = 66° (TIR excitation). Fluorescence images excited at Θ = 62° show some additional red fluorescence due to doxorubicin, which almost disappears at Θ = 66°. Fluorescence spectra excited at Θ = 62° exhibit two emission maxima around 530 nm (GFP) and 543 nm with a long-wave tail, possibly related to doxorubicin or its degradation product. The long-wave part of the spectrum is less pronounced in the TIRFM experiments.

It should be mentioned that some fluorescence of doxorubicin or its degradation product [25] may overlap GFP fluorescence, as proven for whole-cell experiments (upon illumination at Θ = 62°, i.e., below the critical angle of incidence, Θ_C_). As shown in Figure 5, this overlap becomes obvious in the cell nucleus (at Θ = 62°) and is very low in the TIRFM experiments (Θ ≥ 66°). Therefore, fluorescence of doxorubicin or its degradation product probably does not—or only very slightly—falsify our experimental results on cell-substrate distances based on TIRFM.

**Figure 6:**
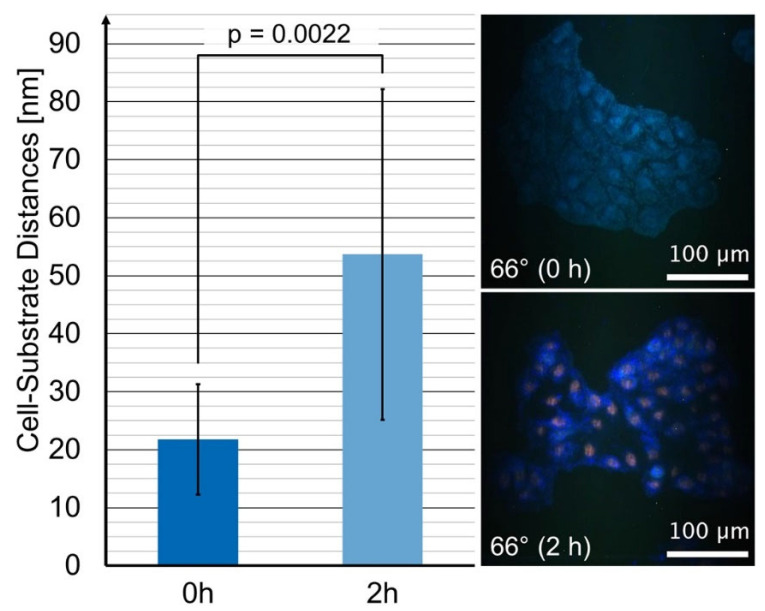
Cell-substrate distances of MCF-7 breast cancer cells incubated with the fluorescence membrane marker laurdan (8 µM, 1 h) prior to and after incubation with doxorubicin (2 µM, 2 h), as evaluated in the spectral maxima at 500–520 nm of VA-TIRFM experiments of *n* = 11 samples in each case. Mean values ± standard deviations including *p*-value for statistical significance (*p* ≤ 0.05: statistically significant). TIRFM images of MCF-7 cells prior to (0 h) and after (2 h) incubation with doxorubicin.

Although variable-angle TIRFM of cell-substrate contacts were reported almost 30 years ago [23,31], since then there have been very few further reports in the literature, e.g., in the authors’ previous publications on nanotopology of cell adhesion to distinguish between tumor cells and less malignant cells [24], and on cell-substrate topology in photodynamic therapy (PDT) [32]. An application of this technique to test the efficacy of doxorubicin (or any other cytostatic drug) is hitherto unknown. Therefore, we suggest considering this method for cell monolayers upon fluorescence staining of cell membranes or transfection with membrane-associated fluorescent proteins. The present CHO-pAcGFP1-Mem cell line appeared appropriate for this purpose. Cell lines from tumors, e.g., breast cancer, bronchial carcinoma, or lymphoma, which have been treated by doxorubicin for many years, are candidates for further studies. Therefore, in a preliminary study (described in the Supplementary Information, Section S3), we tested MCF-7 breast cancer cells prior to and after 2 h incubation with doxorubicin using our Variable-Angle (VA)-TIRFM method. Since cell membranes were nonfluorescent, we stained them with the well-known marker 6-dodecanoyl-2-dimethylamino-naphthalene (laurdan) [33] and evaluated the angular dependence of fluorescence intensity in the spectral band of 500–520 nm. Images served as a control but were not evaluated quantitatively due their low signal-to-noise ratio. Figure 6 proves that, according to our algorithm, cell-substrate distances increased from (22 ± 9) nm to (54 ± 28) nm after 2 h incubation with doxorubicin. This increase was even more pronounced than for CHO-pAcGFP1-Mem cells and suggests again the applicability of our method. However, the distances were generally lower, possibly since MCF-7 cells grow within smaller colonies (islets) with rather strong adhesion to the substrate (see Figure 6).

VA-TIRFM is a sequential method, i.e., images are recorded at about 10 successively increasing angles of light incidence Θ, which are easily adjusted by a stepping motor directly coupled to a light-deflecting mirror [17]. Furthermore, evaluation of cell-substrate distances for all pixels of an image as well as calculation of histograms has to be performed offline based on Equation (2) using, e.g., the MATLAB script described in Section 5.2. This script also permits correction of slight shifts in the x or y directions, which occasionally occur in the course of our experiments. Errors by artifacts, e.g., interference patterns due to grains of dust, which may have an impact on images recorded at certain angles, are corrected manually by eliminating the corresponding image of a series, as reported in [24]. Altogether, each image requires about 2 min for acquisition and 40 s for calculation by the algorithm. Further automation and rapid adjustment using machine learning programs may reduce these time constants, but online topography does not appear to be possible, since various angles of incidence Θ are needed for acquisition of each image series.

In the future, it may be possible to use intrinsic fluorescence or even light-scattering methods instead of fluorescence markers or fluorescent proteins. This would make the method more universal, but further information concerning the cellular location of fluorophores (or scatterers) as well as highly sensitive (e.g., electron multiplying (EM)-CCD) cameras would be needed.

It remains to be proven whether changes of cell-substrate topology may be considered as an early indicator of apoptosis in general. Shrinking and changes of cell morphology upon apoptosis are well-documented in the literature (for reviews, see [34,35]), and light-scattering methods using wavelength dependence [36] or angular resolution [37] have been reported in view of sensing and quantitation. Since these techniques, however, do not prove early cellular responses, molecular sensors for specific proteins, or nucleic acids of cells undergoing apoptosis have been suggested, including sensors for the enzyme caspase-3 frequently activated in tumor cells [38,39,40]. Luo et al. [41] developed a sensor with a cyan fluorescent protein (CFP) fused to a yellow fluorescent protein (YFP) via a caspase-sensitive amino acid peptide (DEVD). This peptide linker is short enough to bring the two fluorescent proteins in close proximity to each other (≤10 nm) to enable nonradiative (“Förster”) Resonance Energy Transfer (FRET [42]) from CFP to YFP. FRET is interrupted due to cleavage of DEVD by caspase-3 during the onset of apoptosis, resulting in pronounced changes of the fluorescence spectra and lifetimes. Using enhanced fluorescent proteins (ECFP, EYFP) and anchoring the sensor in the plasma membrane (pMem-ECFP-DEVD-EYFP) improved its sensitivity and allowed more specific microscopy techniques to be used, e.g., TIRFM for 2-dimensional cell monolayers [43] or Light Sheet Fluorescence Microscopy (LSFM) for 3-dimensional cell cultures [44]. In both cases, the decrease in acceptor (EYFP) fluorescence and increase in donor (ECFP) lifetime upon apoptosis have been well-documented, even before changes of cell morphology became apparent. Imaging of cell-substrate topology may possibly bridge a gap between early detection of apoptosis by a molecular sensor, as reported above, and rather late detection by measuring changes in cell morphology. The topology method reported in this manuscript probably characterizes early changes of cell membranes (see e.g., [45]) and appears promising, since it is biochemically less complicated than the molecular sensor system, and since it can possibly be applied at an earlier stage of apoptosis than measurements of overall changes of cell morphology, e.g., cell shrinking. Some limitations of the technique have been discussed: dependence on the individual cell line, on cell age, and eventually on cultivation time. A further important parameter would be temperature. So far, all measurements were performed at a room temperature of 22–24 °C. However, it is well-known that membrane stiffness and fluidity depend on temperature [30,46], and concomitant changes of cell-substrate distances should be considered. Therefore, maintaining well-defined temperatures in course of our experiments is an important prerequisite.

## 5. Materials and Methods

### 5.1. Cells

Chinese hamster ovary cells transfected with a membrane-associated green fluorescent protein (CHO-pAcGFP1-Mem) were supplied by the Institute of Laser Technology in Medicine and Metrology (ILM), University of Ulm. Cells were seeded at a density of 200 cells/mm^2^ on glass slides and grown for 72 h in quadriPERM cell culture vessels (Guder Labortechnik GmbH, Bad Oeynhausen, Germany) containing RPMI 1640 medium supplemented with 10% fetal calf serum, 1% Penicillin/Streptomycin, and 500 μg/mL Geneticin at 37 °C and 5% CO_2_. Prior to the experiments, cells were incubated for 2 h or 24 h in medium containing 2 µM doxorubicin before rinsing the glass slides with Earle’s Balanced Salt Solution (EBSS) (all purchased from Sigma-Aldrich GmbH, München, Germany). Nonincubated cells (“0 h”) were used as a reference. It should be mentioned that with 24 h incubation, the total growth period was 96 h compared to 72 h at a lower incubation time. For experiments reported in this paper, we preferentially used subcultures with 56–64 cell splittings, when up to 19 measurements (*n* = 13 for 0 h, *n* = 16 for 2 h, and *n* = 19 for 24 h) were performed with 4 measurements of each object slide in different positions. For control experiments, cells were grown for 96 h in culture medium, and cell-substrate distances were determined without incubation and after 2 h incubation with doxorubicin (*n* = 5 measurements each). “Younger” subcultures with 28–35 or 48–50 cell splittings served for a comparison at 0 h and 2 h incubation with *n* = 20 measurements (0 h) or *n* = 18 measurements (2 h) for subcultures 28–35 and *n* = 6 measurements each for subcultures 48–50. 

### 5.2. VA-TIRFM

For Variable-Angle Total Internal Reflection Microscopy (VA-TIRFM), an upright microscope (Axioplan 1, Carl Zeiss, Jena, Germany) was equipped with a condenser unit permitting excitation of the samples under total internal reflection at variable angles Θ and, thus, variable depths d(Θ) of the evanescent electromagnetic field [17]. Polarized light from an argon ion laser (λ = 476 nm; Innova 90, Coherent, Palo Alto, CA, USA) was incident via a single-mode fiber on a hemicylindrical glass prism, which was optically coupled to the object slide containing the cells. The polarization was always perpendicular to the plane of incidence, and angles between Θ = 66° and Θ = 75° were adjusted by a stepping motor (precision: ± 0.15°; for calibration, see [17]). Fluorescence was detected by a 63×/0.90 water immersion objective lens (dipping into the buffer solution that surrounded the cells) and a long pass filter for λ ≥ 510 nm. Fluorescence spectra were recorded by an optical multichannel analyzer (IMD4562, Hamamatsu Photonics, Ichino-Cho, Japan) combined with a purpose-made polychromator, which permitted a spectral resolution of about 10 nm. Corresponding images were recorded by a CCD camera (AxioCam HRm, Carl Zeiss, Jena, Germany) and integrated for up to 2 s. In addition, some color images were recorded with a Canon 500D reflex camera.

**Figure 7:**
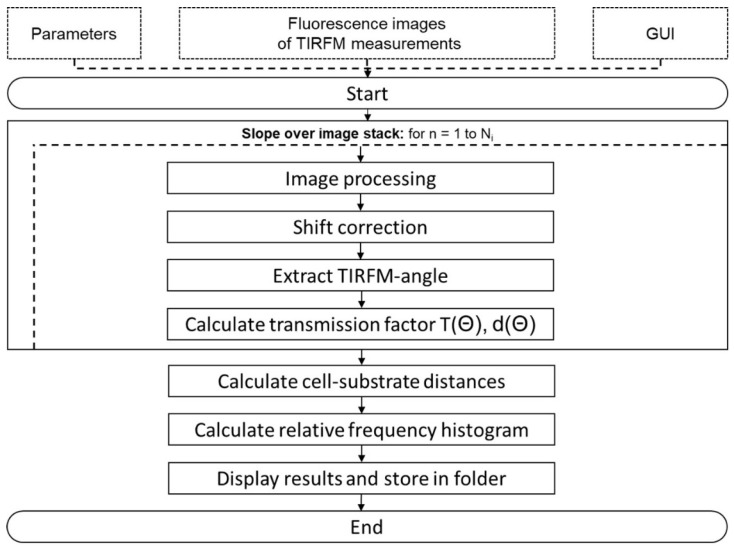
Flow chart of the MATLAB script used for automated cell-substrate calculations (GUI = Graphical User Interface; T(Θ) = transmission factor; d(θ) = penetration depth of the evanescent field). Once the parameters, e.g., the corresponding metric pixel size and the refractive indices, are set and the recorded images are imported into the MATLAB environment, the code gradually starts its calculations. Images are implemented as a virtual stack with Ni corresponding to the maximum number of images for each set. Beginning with a loop covering the image stack, an algorithm is used to correct any possible shifts in the x and y directions, which may occur in individual experiments. Thus, it is ensured that every pixel addresses the same field of view of the recorded cell at various angles. Next, the angle of the recorded image is extracted from the image file name in order to calculate the transmission factor and the penetration depth, as reported above.

The condition for total internal reflection (TIR) was fulfilled for all angles of incidence above the critical angle Θc = arcsin (n_2_/n_1_) = 64.3° with n_1_ = 1.52 corresponding to the refractive index of the glass prism and n_2_ = 1.37 to that of the cytoplasm. This resulted in a penetration depth of the evanescent wave between 72 nm and 167 nm in an angular range 66° ≤ Θ ≤ 75° for a wavelength λ = 476 nm according to Equation (1). While fluorescence spectra served as controls, cell-substrate distances Δ were calculated for all pixels of fluorescence images according to Equation (2) using the automated MATLAB script described in Figure 7. These distances were displayed in a color-coded topology map, from which a relative frequency histogram was calculated. Histograms were determined for all experiments performed under the same conditions (subcultures with 28–35, 48–50, or 56–64 cell splittings as well as incubation times of 0 h, 2 h, or 24 h), and the most frequent distance was evaluated as mean ± standard deviation. A *t*-test for 2 samples assuming unequal variances [47] was applied to check the significance of changes of this distance between 0 h and 2 h as well as between 0 h and 24 h (for subcultures 56–64) upon application of doxorubicin. Changes were regarded as significant at *p* ≤ 0.05.

## Data Availability

Relevant data sets can be found at https://www.hs-aalen.de/users/226 (accessed on 12 February 2022) (“Veröffentlichungen”).

## Supplementary Materials

The following supporting information can be downloaded at: https://www.mdpi.com/article/10.3390/ijms23116277/s1.
