# Supplementary material for: Impact of Doxorubicin on Cell-Substrate Topology
Source: Int J Mol Sci. 2022 Jun 3;23(11):6277. doi: 10.3390/ijms23116277 (PMC9181088; doi:10.3390/ijms23116277)
Supplement: Supplementary file 1 [file ijms-23-06277-s001.zip › ijms-1735987-supplementary.pdf]

Supplementary Material

1. Dependence of fluorescence intensity with increasing TIRF angle

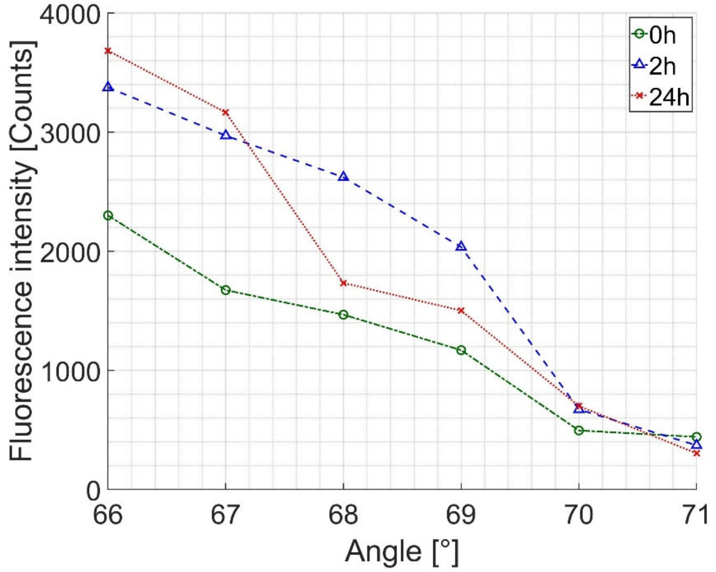

Supplementary Figure S1. Fluorescence intensity measured in the emission maximum (530 nm) of representative samples of CHO-pAcGFP1-Mem cells after 0 h, 2 h, and 24 h incubation with doxorubicin at various TIRF angles (limited to 66° ≤ Θ ≤ 71° for reasons of clarity).

2. Calculation of cell-substrate distances

Absorbed intensity of light (propagating in z-direction) in a thin layer dz: $dI/dz = \varepsilon(\lambda)\ \ln 10\ c(z)\ I(z)$

with $\varepsilon(\lambda)$ = decadic extinction coefficient, $c(z)$ = absorber concentration, $I(z)$ = light intensity.

Absorbed light intensity of an evanescent wave: $dI/dz = \varepsilon(\lambda)\ \ln 10\ c(z)\ I_e\ (n_2/n_1)\ T(\Theta)\ e^{-z/d(\Theta)}$

with I_e = incident intensity, n_2 = refractive index of the cell, n_1 = refractive index of the substrate, $T(\Theta)$ = transmission factor between cell and substrate, $d(\Theta)$ = penetration depth of the evanescent wave.

Fluorescence excited by the evanescent wave: $I_F(\Theta) = \varepsilon(\lambda)\ \ln 10\ \eta\ (\Omega/4\pi)\ I_e\ (n_2/n_1)\ T(\Theta) \int c(z)\ e^{-z/d(\Theta)}\ dz =$ $I_F(\Theta) = A\ T(\Theta) \int c(z)\ e^{-z/d(\Theta)}\ dz$

with η = quantum yield of fluorescence, Ω = solid angle of detection, $A = \varepsilon(\lambda)\ \ln 10\ \eta\ (\Omega/4\pi)\ I_e\ (n_2/n_1)$.

Fluorescence integrated over a thin layer with thickness t and constant concentration c at a distance Δ from the substrate: $I_F = A\ c\ T(\Theta)\ t\ e^{-\Delta/d(\Theta)}$.

Evaluation of $\ln [I_F / T(\Theta)]$ over $1/d(\Theta)$ results in a linear function with the slope $-\Delta$.

As discussed previously [17], the relevant refractive index of the cell is that of the cytoplasm, if the layer t (cell membrane) is very thin, and if the cell-substrate distance Δ is smaller than the wavelength of light.

3. Comparative experiments of MCF-7 breast cancer cells

MCF-7 human breast cancer cells were obtained from Cell Lines Service (CLS No. 00273, Eppelheim, Germany) and grown in DMEM/HAM F-12 medium (No. 9031.1, Carl Roth GmbH, Munich, Germany) supplemented with 10% fetal calf serum and antibiotics at 37 °C and 5% CO_2. Subcultures with 49–52 cell splittings were used for seeding 120 cells/mm^2 on glass slides and growing them for 72 h in quadriPERM cell culture vessels containing cultivation medium. Prior to the microscopy experiments, cells were incubated for 1 h with the fluorescent membrane marker laurdan (8 μM), and part of them was coincubated for 2 h with medium containing 2 μM doxorubicin. Finally, the glass slides were rinsed with Earle's Balanced Salt Solution (EBSS). Cells incubated only with laurdan, but not with doxorubicin ("0 h") were used as a reference.

For Variable-Angle Total Internal Reflection Microscopy (VA-TIRFM), the same setup as described in the manuscript was used together with a super-continuum fiber laser (SuperK EXTREME with SuperK VARIA tunable single-line filter, NKT Photonics, Birkeroed, Denmark) operated at (440 ± 10) nm, using a 40×/ 0.60 long-distance microscope objective lens and a 475-nm long-pass filter for fluorescence detection. This setup permitted to excite laurdan in its longwave absorption tail and to detect the longwave part of its fluorescence [1]. Fluorescence spectra were recorded by an optical multichannel analyzer (IMD4562, Hamamatsu Photonics, Ichino-Cho, Japan) combined with a purpose-made polychromator, which permitted a spectral resolution of about 10 nm. Corresponding images were recorded by a CCD camera (AxioCam MRc, Zeiss, Germany) and integrated for up to 20 seconds. Fluorescence intensity was determined in the spectral band of 500–520 nm upon variation of the angle Θ ($66° \leq \Theta \leq 75°$), and calculation of cell-substrate distances was performed in a similar way as reported for CHO-pAcGFP1-Mem cells (using equations 2 and 3 of the manuscript with $T(\Theta)$ corresponding now to an "effective" transmission factor for unpolarized light [2]). Fluorescence intensity was determined prior to ("0 h") and subsequent to (2 h) incubation with doxorubicin from 11 object fields of 25 μm × 200 μm each, and mean values ± standard deviations (including p-values of statistical significance) were determined as for the CHO-pAcGFP1-Mem cells. The result is documented in Figure 6 of the manuscript and discussed further there. Images recorded for individual angles served as a control, but their angular dependence was not evaluated quantitatively due the low signal-to-noise ratio.

Fluorescence spectra of MCF-7 human breast cancer cells are documented in Supplementary Figure 2 upon whole-cell illumination ($\Theta = 62° \leq \Theta_C$) and TIRFM ($\Theta = 66° \geq \Theta_C$) prior to (0 h) and subsequent to (2 h) incubation with doxorubicin. Upon whole-cell illumination, the longwave laurdan fluorescence is clearly documented, and upon incubation with doxorubicin a strong overlap by its fluorescence or by the fluorescence of its degradation product [3] becomes evident. The TIRFM spectra, however, primarily show the laurdan fluorescence with only very little overlap by doxorubicin. This proves that laurdan is mainly located in the plasma membrane; doxorubicin and its degradation product, however, are inside the cells.

References:

1. Parasassi, T.; de Stasio, G.; d'Ubaldo, A.; Gratton, E. Phase fluctuation in phospholipid membranes revealed by laurdan fluorescence. Biophys. J. 1990, 57 (6), 1179–1186, doi: 10.1016/S0006-3495(90)82637-0.
2. Reichert, W.M.; Truskey, G.A. Total internal reflection fluorescence (TIRF) microscopy. (I) Modelling cell contact region fluorescence. *J. Cell Sci.* **1990**, 96, 219–230, doi: 10.1242/jcs.96.2.219.
3. Hovorka, O.; Šubr, V.; Vetvicka, D.; Kovar, L.; Strohalm, J.; Strohalm, M.; Benda, A.; Hof, M.; Ulbrich, K.; Rihova, B. Spectral analysis of doxorubicin accumulation and the indirect quantification of its DNA intercalation. *Eur. J. Pharm. Biopharm*. **2010**, 76(3), 514–524, doi: 10.1016/j.ejpb.2010.07.008.

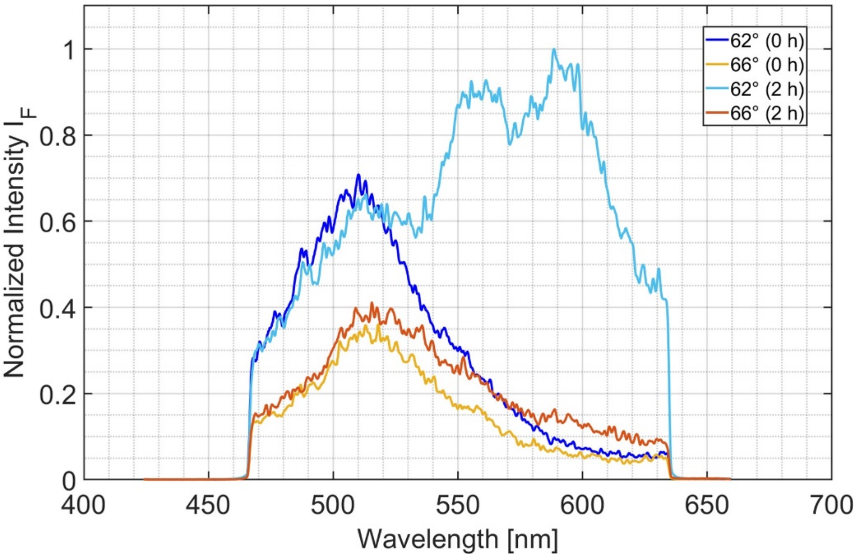

Supplementary Figure S2. Fluorescence spectra of MCF-7 human breast cancer cells incubated with the membrane marker laurdan (8 μM, 1 h) prior to (0 h) and subsequent to (2 h) incubation with doxorubicin (2 μM, 2 h) upon whole-cell illumination (62°) and TIRFM (66°).
